# Ectopic Fat Accumulation and Distant Organ-Specific Insulin Resistance in Japanese People with Nonalcoholic Fatty Liver Disease

**DOI:** 10.1371/journal.pone.0092170

**Published:** 2014-03-20

**Authors:** Ken-ichiro Kato, Toshinari Takamura, Yumie Takeshita, Yasuji Ryu, Hirofumi Misu, Tsuguhito Ota, Kumpei Tokuyama, Shoichiro Nagasaka, Munehide Matsuhisa, Osamu Matsui, Shuichi Kaneko

**Affiliations:** 1 Department of Disease Control and Homeostasis, Kanazawa University Graduate School of Medical Sciences, Kanazawa, Ishikawa, Japan; 2 Department of Radiology, Kanazawa University Graduate School of Medical Sciences, Kanazawa, Ishikawa, Japan; 3 Graduate School of Comprehensive Human Science, University of Tsukuba, Tsukuba, Ibaraki, Japan; 4 Department of Medicine, Division of Endocrinology and Metabolism, Jichi Medical University, Shimono, Tochigi, Japan; 5 Clinical Research Center for Diabetes, Tokushima University, Tokushima, Tokushima, Japan; Northeast Ohio Medical University, United States of America

## Abstract

**Objective:**

The aim of this study was to examine the association between ectopic fat and organ-specific insulin resistance (IR) in insulin-target organs in patients with nonalcoholic fatty liver disease (NAFLD).

**Methods:**

Organ-specific IR in the liver (hepatic glucose production (HGP)×fasting plasma insulin (FPI) and suppression of HGP by insulin [%HGP]), skeletal muscle (insulin-stimulated glucose disposal [Rd]), and adipose tissue (suppression of FFA by insulin [%FFA]) was measured in 69 patients with NAFLD using a euglycemic hyperinsulinemic clamp with tracer infusion ([6,6-^2^H_2_]glucose). Liver fat, intramyocellular lipid (IMCL), and body composition were measured by liver biopsy, proton magnetic resonance spectroscopy, and bioelectrical impedance analysis, respectively.

**Results:**

HGP×FPI was significantly correlated with Rd (*r* = −0.57, *P*<0.001), %HGP with %FFA (*r* = 0.38, *P*<0.01), and Rd with %FFA (*r* = 0.27, *P*<0.05). Liver steatosis score was negatively associated with Rd (*r* = −0.47, *P*<0.001) as well as with HGP×FPI (*r = *0.43, *P*<0.001). Similarly, intrahepatic lipid was negatively associated with Rd (*r* = −0.32, *P*<0.05). IMCL was not associated with Rd (*r* = −0.16, *P* = 0.26). Fat mass and its percentage were associated with HGP×FPI (*r = *0.50, *P*<0.001; *r = *0.48, *P*<0.001, respectively) and Rd (*r* = −0.59, *P*<0.001; *r* = −0.52, *P*<0.001, respectively), but not with %FFA (*r* = −0.21, *P* = 0.10; *r* = −0.001, *P* = 0.99, respectively).

**Conclusion:**

Unexpectedly, fat accumulation in the skeletal muscle and adipose tissue was not associated with organ-specific IR. Instead, liver fat was associated not only with hepatic IR but also with skeletal muscle IR, suggesting a central role of fatty liver in systemic IR and that a network exists between liver and skeletal muscle.

## Introduction

Insulin resistance (IR) is a core pathology of type 2 diabetes mellitus (T2DM), nonalcoholic fatty liver disease (NAFLD), and cardiovascular diseases [1]–[3]. The severity of IR may differ among the major insulin-target organs, the liver, skeletal muscle, and adipose tissue [4]. Accumulating evidence suggests that ectopic fat accumulation in insulin-target organs leads to development of IR in each organ by altering oxidative stress [5]–[7] and gene expression profiles [8], [9]. Indeed, liver steatosis is associated with whole-body IR, independently of body mass index (BMI) [10].

Conversely, inter-organ network and organ-derived bioactive hormones such as adiponectin and selenoprotein P may play a role in the development of distant organ IR [11]–[13]. Therefore, to understand organ networks that sense excess energy and regulate insulin action, elucidating the association between fat accumulation and organ-specific IR among the liver, skeletal muscle, and adipose tissue is important, especially in humans. However, no previous studies have demonstrated the association among these organs comprehensively and simultaneously [14], [15]. In addition, liver biopsy remains gold standard for diagnosis of NAFLD because it more accurately measures liver fat than proton magnetic resonance spectroscopy (^1^H-MRS) under some conditions [16].

The present study try to address the association of organ-specific IR with ectopic fat among the liver, skeletal muscle, and adipose tissue in Japanese patients with NAFLD, systematically using reliable methods including liver biopsy, assessment of glucose metabolism measured by a euglycemic hyperinsulinemic clamp study with stable-isotope, and ^1^H-MRS.

## Materials and Methods

### Ethics Statement

The study was approved by the Medical Ethics Committee of Kanazawa University (Approval No. 845), and written informed consent was obtained from each patient prior to participation. The study was conducted in accordance with the Declaration of Helsinki.

### Participants and Study Design

We studied 69 patients clinically diagnosed with NAFLD, recruited consecutively between 2010 and 2012 from Kanazawa University Hospital, Japan. The patients were in good general health without evidence of any acute or chronic diseases (other than NAFLD, T2DM, hypertension, or dyslipidemia) as determined by history, physical examination, routine blood chemistries, urinalysis, and electrocardiography. Out of the 69 patients, 37 (54%) had T2DM according to the American Diabetes Association criteria. Of the 37 T2DM patients, antidiabetic agents were administered to 18 patients in monotherapy and 7 patients in combination therapy (metformin, n = 15; dipeptidyl peptidase-4 inhibitors, n = 9; glucagon-like peptide-1 agonists, n = 7; mealtime dosing of a rapid-acting insulin analog, n = 5, respectively). None of the patients were taking α-glucosidase inhibitors, rapid-acting insulin secretion agents, sulfonylurea, thiazolidinediones, or long-acting insulin. Participants were excluded if they had a history of alcohol abuse (more than 20 g/day), liver diseases other than NAFLD (hepatitis B or C, autoimmune hepatitis, hemochromatosis, Wilson disease, drug-induced disease, or other), type 1 diabetes, or a history of clinically significant renal, pulmonary, or heart diseases.

The participants were studied on four separate occasions. Generally, all measurements were performed within 1 month and included: 1) organ-specific IR in the liver, skeletal muscle, and adipose tissue by a euglycemic hyperinsulinemic clamp study with tracer ([6,6-^2^H_2_]glucose) infusion; 2) liver biopsy for histology to confirm the diagnosis of NAFLD and score the degree of steatosis, grade, and stage; 3) intrahepatic lipid (IHL) and intramyocellular lipid (IMCL) measured by ^1^H-MRS, and body composition by a bioelectrical impedance analysis; and 4) 75-g oral glucose tolerance test (OGTT) to evaluate the glucose tolerance according to American Diabetes Association criteria [17].

### Euglycemic Hyperinsulinemic Clamp

After an overnight fast, two intravenous catheters, one for blood sampling and one for infusion of glucose, insulin, and tracers, were inserted in the antecubital vein of each arm. At 0700 h, after obtaining a blood sample for background enrichment of plasma glucose, a continuous infusion of [6,6-^2^H_2_]glucose (>99% enriched; Cambridge Isotope, Andover, MA, USA) was started at a rate of 0.05 mg·kg^−1^·min^−1^ after a priming dose equivalent. After 100, 110, and 120 min, blood samples were obtained for determination of tracer enrichments. Subsequently, at 0900 h, the euglycemic hyperinsulinemic clamp study was started using an artificial pancreas (model STG-55; Nikkiso, Tokyo, Japan), as described previously [18], [19]. A primed continuous infusion of insulin (Humulin R; Eli Lilly, Indianapolis, IN, USA) was started for 2.0 h at a rate of 1.25 mU·kg^−1^·min^−1^ to attain a plasma insulin concentration of approximately 100 μU/mL. Glucose was infused to maintain a plasma glucose concentration of 100 mg/dL (or 90 mg/dL for baseline values under 90 mg/dL). Simultaneously, [6,6-^2^H_2_]glucose infusion was continued at a rate of 0.15 mg·kg^−1^·min^−1^. During the last 20 min of the clamp study, blood samples were obtained in 10-min intervals to determine tracer enrichments.

### Liver Biopsy/Pathology

Ultrasound-guided liver biopsy specimens were obtained from all 69 patients. Each specimen was stained with hematoxylin-eosin and silver reticulin stains and histologically examined by one experienced pathologist who was blinded to the patient’s clinical condition and biochemical data. The biopsied tissues were scored for steatosis (0, none; 1, <33%; 2, 33–66%; 3, >66%), stage, and grade as described previously (10), according to the standard criteria for grading and staging of nonalcoholic steatohepatitis proposed by Brunt et al. [20], [21].

### Liver Fat Content and IMCL (Proton Magnetic Resonance Spectroscopy)

IHL and IMCL were measured as reported previously [22], [23]. Briefly, IHL of the liver’s right lobe and IMCL of the soleus muscle were measured by ^1^H-MRS using a whole-body 3.0 T MR System (Signa HDxt 3.0 T, General Electric Healthcare, Milwaukee, WI, USA). Voxels (3.0×3.0×3.0 cm^3^ for liver and 2.0×2.0×2.0 cm^3^ for soleus muscle) were positioned in the liver or soleus muscle to avoid blood vessels and visible interfacial fat, and the voxel sites were carefully matched at each examination. Imaging parameters were set to repetition time of 1500 ms and echo time of 27 ms. To quantify IHL and IMCL, the MR spectral raw data were processed by using the LCModel software (Version 6.3-0C, Stephen Provencher, Oakville, Ontario, Canada).

### Body Composition

Body composition, such as fat mass and fat-free mass, was determined by a bioelectrical impedance analysis (Tanita BC-118D, Tanita, Tokyo, Japan).

### Oxygen Consumption

Oxygen consumption was measured using indirect calorimetry (Aeromonitor AE310S, Minato, Osaka, Japan).

### 75-g OGTT

After an overnight fast, a 75-g OGTT was performed at 0800 h. Blood samples were collected at 0, 30, 60, 90, 120, and 180 min to measure plasma glucose insulin and C-peptide concentrations.

### Analytical Methods

Plasma glucose was measured by the glucose oxidase method (Glucose Analyzer GA09; A&T, Kanagawa, Japan), and plasma insulin and C-peptide were measured using a sandwich enzyme immunoassay system with E-test Tosoh II (IRI) and E-test Tosoh II (C-peptide) (Tosoh, Tokyo, Japan). Plasma FFA was measured by a standard colorimetric method using NEFA-SS (Eiken, Tokyo, Japan). Hemoglobin A1c level was measured using high-performance liquid chromatography (TOSOH HLC-723G8; Tosoh, Tokyo, Japan).

Deuterated glucose was analyzed as a penta-acetate derivative using the method by Wolfe [24]. Samples were analyzed on a quadrupole gas chromatography mass spectrometry instrument (GCMS-QP1100EX, Shimadzu, Kyoto, Japan) operated in the electron impact mode by selective-ion monitoring of m/z 200, 201, and 202. Oven temperature was 180°C with a 10°C/min rate of temperature rise until 250°C with a 25 m HR-1 capillary column (Shinwa Chemical Industries, Kyoto, Japan). Tracer concentrations were calculated based on the sample’s tracer-to-tracee mass ratio [25].

### Calculations

In the basal state, hepatic glucose production (HGP) was calculated as the rate of appearance (Ra) of glucose according to the Steele’s equation as previously described [19], [26]. During the clamp study, glucose Ra was calculated using Steele’s equation from tracer data [26]. HGP during the clamp study was calculated as the difference between glucose Ra and the infusion rate of exogenous glucose.

We calculated and defined organ-specific IR in the liver, skeletal muscle, and adipose tissue as described previously [27]–[30]. Hepatic IR indices were calculated as the product of fasting HGP and fasting plasma insulin (FPI) concentration (HGP×FPI [(mg·kg^−1^·min^−1^)×(μU/mL)]) and suppression of HGP by insulin during a clamp study (%HGP). The skeletal muscle IR index was calculated as insulin-stimulated glucose disposal (Rd), and the adipose tissue IR index was calculated as suppression of FFA by insulin during a clamp study (%FFA).

### Statistical Analysis

All analyses were performed using SPSS software version 21.0 (SPSS Inc., Chicago, IL, USA). All values are expressed as mean ± SEM, unless stated otherwise. The relationship between individual variables was assessed by Pearson’s correlation for parametric variables and by Spearman’s correlation for non-parametric variables. Multiple linear regression analysis was used to assess independent determinants of organ-specific IR. The differences between the two groups were assessed by Student’s *t*-test for continuous variables and chi-square test for categorical variables. Data involving more than two groups were assessed by analysis of variance (ANOVA). Statistical significance was considered to be *P*<0.05.

## Results

### Organ-specific IR and Clinical Characteristics in Patients with NAFLD

The characteristics of the study subjects and their metabolic profiles are shown in Table 1. During the clamp study, plasma glucose concentrations were maintained at baseline values (103±1 mg/dL; mean ± SEM), and steady-state plasma insulin concentrations were reached at 110.2±3.6 μU/mL. Basal HGP was 2.09±0.08 mg·kg^−1^·min^−1^ in subjects with normal glucose tolerance (NGT), 2.18±0.10 mg·kg^−1^·min^−1^ in subjects with impaired glucose tolerance (IGT) and 2.67±0.12 mg·kg^−1^·min^−1^ in subjects with T2DM. Rd was 3.81±0.18 mg·kg^−1^·min^−1^ in NGT, 3.27±0.17 mg·kg^−1^·min^−1^ in IGT and 3.57±0.14 mg·kg^−1^·min^−1^ in T2DM. Basal FFA was 0.47±0.05 mEq/L in NGT, 0.56±0.04 mEq/L in IGT and 0.60±0.04 mEq/L in T2DM. Basal HGP showed a significant positive correlation with fasting plasma glucose levels (*r* = 0.48, *P*<0.001). Rd showed a significant positive correlation with basal oxygen consumption rate per body weight (VO_2_) (*r* = 0.42, *P*<0.01). FFA and HGP were suppressed from baseline by 77.0±1.4% and 69.3±2.8%, respectively. These values are similar to previous data in Japanese [31] and European descent [1], [27], [29] subjects.

**Table 1:** Clinical characteristics of the study subjects.

	All	non-DM (NGT+IGT)	T2DM	*P* value*
*n*	69	32	37	
Age (years)	51±2	46±3	55±2	0.008^b^
Sex (Male/Female)	42/27	23/9	19/18	0.082
Body mass index (kg/m^2^)	30.3±0.9	30.9±1.2	29.8±1.4	0.526
Weight (kg)	82.3±2.7	86.5±3.8	78.6±3.9	0.152
Fat-free mass (kg)	50.2±1.3	52.7±1.6	47.9±1.9	0.058
Total fat mass (kg)	30.6±2.0	31.0±2.9	30.2±2.9	0.855
Body fat percentage (%)	36.3±1.3	35.6±1.9	37.0±1.8	0.594
Historogical scores				
Steatosis (0/1/2/3)	5/33/15/16	4/13/5/10	1/20/10/6	
Grade (0/1/2/3)	15/35/16/3	10/14/7/1	5/21/9/2	
Stage (0/1/2/3/4)	20/29/6/11/3	12/14/1/4/1	8/15/5/7/2	
NAFLD activity score (0/1/2/3/4/5/6/7/8)	3/5/12/14/12/9/13/1/0	2/4/4/7/4/5/5/1/0	1/1/8/7/8/4/8/0/0	
IHL (mmol/L)	9.63±1.01	7.30±1.27	11.23±1.41	0.056
IMCL (AU, ratio relative to creatine)	28.29±1.49	27.91±2.21	28.58±2.04	0.827
Glucose tolerance (NGT/IGT/DM)	11/21/37			
Hemoglobin A1C (%)	6.5±0.1	6.0±0.1	7.0±0.1	<0.001^c^
Fasting plasma glucose (mg/dL)	111±2	100±2	121±3	<0.001^c^
2-h glucose (mg/dL)	208±10	142±6	265±12	<0.001^c^
Fasting plasma insulin (μU/mL)	14.0±1.0	15.1±1.5	13.1±1.3	0.318
2-h insulin (μU/mL)	139.1±12.6	157.2±21.8	123.5±13.7	0.182
Insulinogenic index [(μU/mL)/(mg/dL)]	0.72±0.09	0.95±0.16	0.52±0.09	0.019^a^
Fasting C-peptide (ng/mL)	2.9±0.1	3.1±0.2	2.7±0.2	0.199
Fasting FFAs (mEq/L)	0.57±0.03	0.53±0.03	0.60±0.04	0.254
Total cholesterol (mg/dL)	176±4	185±6	169±6	0.061
Triglycerides (mg/dL)	153±11	150±10	155±18	0.801
HDL cholesterol (mg/dL)	41±1	41±2	41±2	0.927
Aspartate aminotransferase (IU/L)	37±2	37±3	37±3	0.969
Alanine aminotransferase (IU/L)	60±4	62±6	59±5	0.752
Basal HGP (mg·kg^−1^·min^−1^)	2.43±0.08	2.15±0.07	2.67±0.12	0.001^b^
HGP×FPI [(mg·kg^−1^·min^−1^)×(μU/mL)]	32.0±2.0	31.2±2.8	32.7±2.9	0.707
Euglycemic hyperinsulinemic clamp [clamp period]				
Clamped glucose (mg/dL)	103±1	102±2	104±2	0.592
Steady state plasma insulin (μU/mL)	110.2±3.6	115.7±6.0	105.3±4.2	0.164
FFAs (mEq/L)	0.13±0.01	0.13±0.01	0.13±0.01	0.884
%FFA (%)	77.0±1.4	75.9±2.0	78.0±1.9	0.455
HGP (mg·kg^−1^·min^−1^)	0.69±0.07	0.56±0.07	0.81±0.12	0.088
%HGP (%)	69.3±2.8	73.4±3.5	65.8±4.1	0.170
Rd (mg·kg^−1^·min^−1^)	3.52±0.10	3.45±0.14	3.57±0.14	0.556
Rd/SSPI [(mg·kg^−1^·min^−1^)/(μU/mL)]	0.035±0.002	0.033±0.003	0.037±0.003	0.332
VO_2_ (ml·kg^−1^·min^−1^)	2.85±0.04	2.84±0.07	2.86±0.06	0.817

Data are presented as n or mean ± SEM.

IHL, intrahepatic lipid; IMCL, intramyocellular lipid; AU, arbitrary units; HGP, hepatic glucose production; FPI, fasting plasma insulin; SSPI, steady state plasma insulin; VO_2_, basal oxygen consumption rate per body weight.

*Difference between the non-DM group and the T2DM group.

a *P*<0.05,

b *P*<0.01,

c *P*<0.001.

The relationship between clinical characteristics and organ-specific insulin sensitivity/resistance indices is shown in Table 2. HGP×FPI was significantly correlated with Rd (*r* = −0.57, *P*<0.001), %HGP with %FFA (*r* = 0.38, *P*<0.01), and Rd with %FFA (*r* = 0.27, *P*<0.05) suggesting that the IRs in the liver, skeletal muscle, and adipose tissue were significantly associated with each other, although the correlation was not very strong.

**Table 2:** Univariate correlation between ectopic fat and organ-specific insulin resistance.

	HGP×FPI		%HGP		Rd		Rd/SSPI		%FFA	
	*r*	*P*	*r*	*P*	*r*	*P*	*r*	*P*	*r*	*P*
HGP×FPI	1	−	−0.130	0.288	−0.574^c^	<0.001	−0.489^c^	<0.001	−0.168	0.167
%HGP	−0.130	0.288	1	−	0.167	0.170	0.387^b^	0.001	0.375^b^	0.002
Rd	−0.574^c^	<0.001	0.167	0.170	1	−	0.766^c^	<0.001	0.272^a^	0.024
Rd/SSPI	−0.489^c^	<0.001	0.387^b^	0.001	0.766^c^	<0.001	1	−	0.296^a^	0.014
%FFA	−0.168	0.167	0.375^b^	0.002	0.272^a^	0.024	0.296^a^	0.014	1	−
Steatosis	0.428^c^	<0.001	−0.148	0.226	−0.473^c^	<0.001	−0.430^c^	<0.001	−0.121	0.322
Grade	0.338^b^	0.004	−0.111	0.362	−0.376^b^	0.001	−0.338^b^	0.005	−0.055	0.656
Stage	0.283^a^	0.019	−0.098	0.422	−0.348^b^	0.003	−0.261^a^	0.031	−0.010	0.933
IHL	0.245	0.089	−0.114	0.436	−0.315^a^	0.028	−0.271	0.062	−0.135	0.356
IMCL	0.250	0.065	−0.215	0.115	−0.156	0.256	−0.183	0.185	−0.060	0.662
Fat-free mass	0.031	0.801	−0.117	0.347	−0.216	0.079	−0.211	0.090	−0.433^c^	<0.001
Total fat mass	0.495^c^	<0.001	−0.147	0.235	−0.594^c^	<0.001	−0.536^c^	<0.001	−0.205	0.096
Body fat percentage	0.481^c^	<0.001	−0.115	0.355	−0.518^c^	<0.001	−0.478^c^	<0.001	−0.001	0.994
VO_2_	−0.129	0.342	0.191	0.158	0.418^b^	0.001	0.405^b^	0.002	0.115	0.397

HGP, hepatic glucose production; FPI, fasting plasma insulin; SSPI, steady state plasma insulin; IHL, intrahepatic lipid; IMCL, intramyocellular lipid; VO_2_, basal oxygen consumption rate per body weight.

a *P*<0.05,

b *P*<0.01,

c *P*<0.001.

### Ectopic Fat and Organ-specific IR

Histological liver steatosis score was strongly correlated with IHL measured by ^1^H-MRS (*r = *0.75, *P*<0.001).

**Figure 1:**
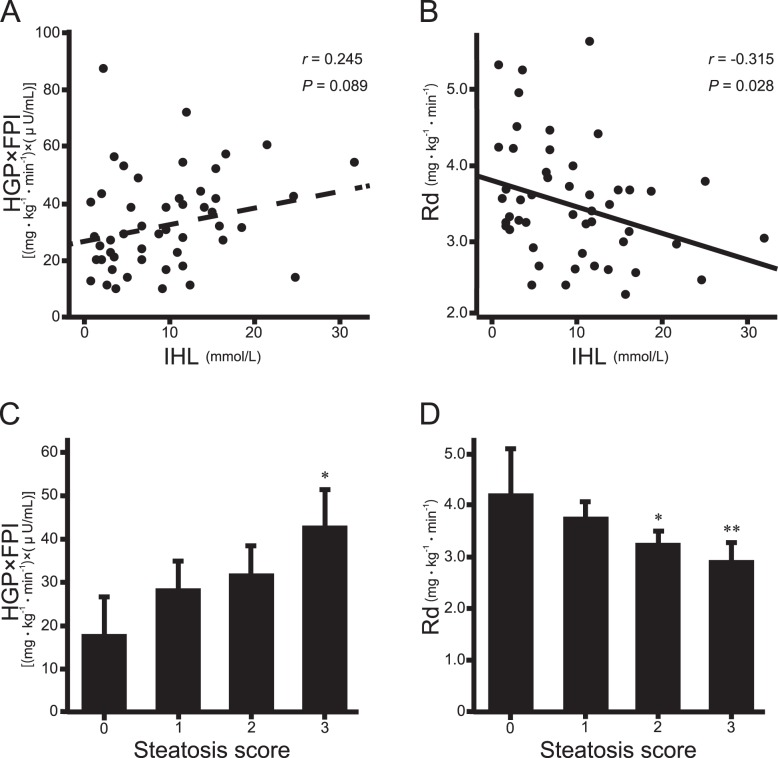
Correlation between liver fat and organ-specific insulin resistance (IR). (A) univariate correlation between IR in the liver (HGP×FPI) and liver fat (IHL) (*r = *0.25, *P* = 0.09). (B) univariate correlation between skeletal muscle IR index (Rd) and liver fat (IHL) (*r* = −0.32, *P*<0.05). (C) IR in the liver (HGP×FPI) stratified by steatosis score. (D) skeletal muscle IR index (Rd) stratified by steatosis score. *P<0.05 vs. score 0 steatosis group. **P<0.01 vs. score 0 steatosis group.

Liver steatosis score was significantly correlated with Rd (*r* = −0.47, *P*<0.001) as well as HGP×FPI (*r = *0.43, *P*<0.001) (Table 2). Similarly, IHL was significantly correlated with Rd (*r* = −0.32, *P*<0.05) and tended to be correlated with HGP×FPI (*r = *0.25, *P* = 0.09) (Figure 1A, 1B). In the multiple regression analysis, liver steatosis score was significantly correlated with both HGP×FPI (β = 0.284, *P*<0.05) and Rd (β = −0.300, *P*<0.01) after adjusting for age, sex, and BMI. Correlation of liver steatosis score with Rd (β = −0.261, *P*<0.05) was significant after further adjusting for total fat mass (Table 3). When stratified by steatosis score, HGP×FPI was significantly higher and Rd was significantly lower in the score 3 steatosis group compared to the score 0 steatosis group (*P*<0.01; *P*<0.001, respectively) (Figure 1C,1D).

**Table 3:** Multiple regression models predicting HGP×FPI and Rd.

	HGP×FPI		Rd	
	β	*P*	β	*P*
Steatosis (Model 1)	0.284	0.026^a^	−0.300	0.007^b^
Steatosis (Model 2)	0.216	0.098	−0.261	0.027^a^

HGP, hepatic glucose production; FPI, fasting plasma insulin.

Model 1, adjusted for, age, sex, and body mass index; Model 2, adjusted for, age, sex, body mass index, and total fat mass.

a *P*<0.05,

b *P*<0.01.

**Figure 2:**
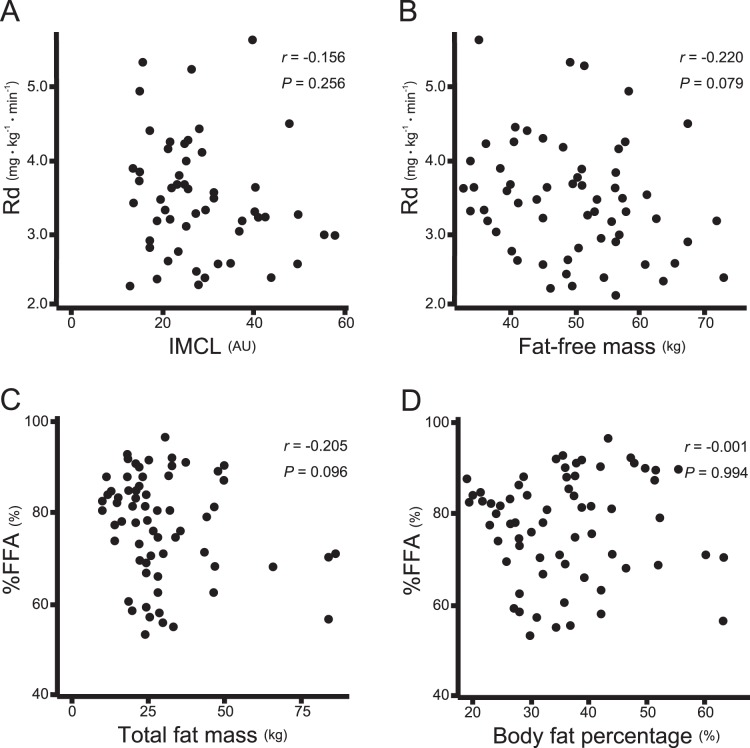
Correlation between ectopic fat and organ-specific insulin resistance (IR). (A) univariate correlation between skeletal muscle IR index (Rd) and intramyocellular lipid (IMCL) (*r* = −0.16, *P* = 0.26). (B) univariate correlation between Rd and fat-free mass (*r = *0.22, *P* = 0.08). (C) univariate correlation between adipose tissue IR index (%FFA) and total fat mass (*r* = −0.21, *P* = 0.10). (D) univariate correlation between %FFA and body fat percentage (*r* = −0.00, *P* = 0.99).

Unexpectedly, indices of fat accumulation in the skeletal muscle (IMCL) and adipose tissue were not associated with their own organ-specific IR (Table 2). IMCL and fat-free mass were not correlated with Rd (*r* = −0.16, *P* = 0.26; *r* = −0.22, *P* = 0.08, respectively) (Figure 2A,2B). Total fat mass and its percentage were correlated with HGP×FPI (*r = *0.50, *P*<0.001; *r = *0.48, *P*<0.001, respectively) and Rd (*r* = −0.59, *P*<0.001; *r* = −0.52, *P*<0.001, respectively), but not with %FFA (*r* = −0.21, *P* = 0.10; *r* = −0.00, *P* = 0.99, respectively) (Figure 2C,2D).

Similar results were obtained when Rd was normalized by steady state plasma insulin (Rd/SSPI) (Table 2).

Because it may be possible that T2DM itself is associated with IR independently with organ steatosis, we analyzed the subjects with or without T2DM. Age, hemoglobin A1c, fasting plasma glucose, 2-h glucose level of 75-g OGTT and basal HGP were significantly higher in T2DM group compared to non-DM group (Table 1). Regardless of the presence or absence of T2DM, liver steatosis score was significantly correlated with Rd as well as HGP×FPI, and IMCL and total fat mass were not correlated with Rd or %FFA respectively (Table 4, Table 5). The results of the multiple regression analysis are shown in Table S1 and Table S2.

**Table 4:** Univariate correlation between ectopic fat and organ-specific insulin resistance in subjects without type 2 diabetes (*n* = 32).

	HGP×FPI		%HGP		Rd		Rd/SSPI		%FFA	
	*r*	*P*	*r*	*P*	*r*	*P*	*r*	*P*	*r*	*P*
HGP×FPI	1	−	−0.132	0.470	−0.551^b^	0.001	−0.554^b^	0.001	−0.095	0.607
%HGP	−0.132	0.470	1	−	0.101	0.583	0.121	0.509	0.370^a^	0.037
Rd	−0.551^b^	0.001	0.101	0.583	1	−	0.798^c^	<0.001	0.297	0.099
Rd/SSPI	−0.554^b^	0.001	0.121	0.509	0.798^c^	<0.001	1	−	0.210	0.250
%FFA	−0.095	0.607	0.370^a^	0.037	0.297	0.099	0.210	0.250	1	−
Steatosis	0.524^b^	0.002	−0.008	0.964	−0.478^b^	0.006	−0.409^a^	0.020	0.099	0.589
Grade	0.403^a^	0.022	−0.115	0.530	−0.487^b^	0.005	−0.497^b^	0.004	0.172	0.348
Stage	0.206	0.258	−0.033	0.859	−0.305	0.090	−0.194	0.286	0.114	0.534
IHL	0.198	0.403	0.155	0.515	−0.570^b^	0.009	−0.414	0.070	−0.173	0.465
IMCL	0.318	0.130	−0.334	0.110	−0.128	0.552	−0.151	0.480	−0.172	0.421
Fat-free mass	−0.017	0.926	−0.262	0.154	−0.050	0.788	−0.118	0.527	−0.321	0.078
Total fat mass	0.523^b^	0.003	0.030	0.873	−0.728^c^	<0.001	−0.586^b^	0.001	−0.082	0.660
Body fat percentage	0.488^b^	0.005	0.056	0.763	−0.729^c^	<0.001	−0.599^c^	<0.001	0.045	0.811
VO_2_	−0.045	0.829	0.131	0.523	0.379	0.057	0.356	0.074	0.009	0.964

HGP, hepatic glucose production; FPI, fasting plasma insulin; SSPI, steady state plasma insulin; IHL, intrahepatic lipid; IMCL, intramyocellular lipid; VO_2_, basal oxygen consumption rate per body weight.

a *P*<0.05,

b *P*<0.01,

c *P*<0.001.

**Table 5:** Univariate correlation between ectopic fat and organ-specific insulin resistance in subjects with type 2 diabetes (*n* = 37).

	HGP×FPI		%HGP		Rd		Rd/SSPI		%FFA	
	*r*	*P*	*r*	*P*	*r*	*P*	*r*	*P*	*r*	*P*
HGP×FPI	1	−	−0.119	0.483	−0.600^c^	<0.001	−0.461^b^	0.005	−0.234	0.164
%HGP	−0.119	0.483	1	−	0.233	0.165	0.582^c^	<0.001	0.419^a^	0.010
Rd	−0.600^c^	<0.001	0.233	0.165	1	−	0.746^c^	<0.001	0.244	0.145
Rd/SSPI	−0.461^b^	0.005	0.582^c^	<0.001	0.746^c^	<0.001	1	−	0.346^a^	0.039
%FFA	−0.234	0.164	0.419^a^	0.010	0.244	0.145	0.346^a^	0.039	1	−
Steatosis	0.390^a^	0.017	−0.306	0.066	−0.459^b^	0.004	−0.486^b^	0.003	−0.340^a^	0.039
Grade	0.263	0.115	−0.023	0.894	−0.282	0.091	−0.216	0.206	−0.304	0.067
Stage	0.357^a^	0.030	−0.096	0.571	−0.478^b^	0.003	−0.473^b^	0.004	−0.168	0.320
IHL	0.286	0.133	−0.122	0.529	−0.209	0.277	−0.219	0.262	−0.197	0.306
IMCL	0.202	0.276	−0.128	0.491	−0.178	0.338	−0.220	0.243	0.025	0.893
Fat-free mass	0.084	0.626	−0.136	0.430	−0.314	0.062	−0.276	0.108	−0.508^b^	0.002
Total fat mass	0.478^b^	0.003	−0.301	0.074	−0.493^b^	0.002	−0.497^b^	0.002	−0.305	0.071
Body fat percentage	0.473^b^	0.004	−0.227	0.183	−0.362^a^	0.030	−0.374^a^	0.027	−0.049	0.775
VO_2_	−0.211	0.264	0.264	0.159	0.460^a^	0.011	0.460^a^	0.012	0.212	0.261

HGP, hepatic glucose production; FPI, fasting plasma insulin; SSPI, steady state plasma insulin; IHL, intrahepatic lipid; IMCL, intramyocellular lipid; VO_2_, basal oxygen consumption rate per body weight.

a *P*<0.05,

b *P*<0.01,

c *P*<0.001.

## Discussion

We comprehensively and simultaneously evaluated ectopic fat accumulation and organ-specific IR in insulin-target organs in Japanese people with NAFLD, and found the following: 1) the IRs in the liver, skeletal muscle, and adipose tissue were associated with each other, 2) indices of fat accumulation in the skeletal muscle and adipose tissue were not associated with their own organ-specific IR, and 3) liver fat was associated with skeletal muscle IR as well as hepatic IR, independently of age, sex, BMI and total fat mass (Figure S1).

Although the IRs in the liver, skeletal muscle, and adipose tissue were associated with each other, the relation was relatively weak. There are a couple possible explanations for this result. First, the main site and the severity of IR may vary among organs and individuals [4]. Second, possibly the %HGP and %FFA are not completely suitable for indices of hepatic and adipose tissue IR, respectively, and might not fully exhibit inter-individual variation because HGP and lipolysis appeared to be more sensitive to suppression by insulin compared to stimulation of Rd by insulin [32], [33]. Lowering steady-state insulin levels by a reduced insulin infusion rate might improve the specificity of these indices to reflect organ insulin sensitivity.

In the present study, IMCL was not associated with skeletal muscle IR. The participants in this study had a wide BMI range (21.3–54.9 kg/m^2^) and subjects may have different physical exercise habits with various intensities. IMCL is increased not only by obesity but also by enhanced physical fitness [34]. Therefore, absolute fat contents do not always predict IR in the skeletal muscle, thus, toxic lipids that cause IR in the skeletal muscle should be further researched. Similarly, we failed to find any relationship between fat mass or its percentage and adipose tissue IR. Although we evaluated only total fat mass, distribution of adipose tissue may potentially determine insulin action. Indeed, visceral fat, but not subcutaneous fat, is reported to be associated with %FFA [35]. Therefore, future studies should evaluate visceral and subcutaneous fat masses separately and evaluate the relation to %FFA in Japanese people.

In addition to the previously well-recognized relationship between adipose tissue mass and IR in the liver and skeletal muscle [36], the present study showed a distinct relationship between liver fat and skeletal muscle IR independently of age, sex, and BMI. Although our results are consistent with previous studies showing that liver fat plays an important role in peripheral IR as well as hepatic IR [14], [15], not all associations among components of ectopic fat and organ-specific IR were examined simultaneously in these studies. Our findings suggest that hepatic steatosis *per se* is a central surrogate pathology indicative of IR in both liver and skeletal muscle in patients with NAFLD. In addition, there may be a network between the liver and skeletal muscle to maintain whole body energy homeostasis. Accordingly, whether hepatic steatosis is a consequence or cause of skeletal muscle IR remains uncertain because a longitudinal observation of the relationship is lacking. One hypothesis is that skeletal muscle IR causes obesity and subsequent hepatic steatosis as experimentally shown in mice with muscle-selective IR [37]. Indeed, Flannery et al. recently reported that skeletal muscle IR promotes increased hepatic *de novo* lipogenesis and hepatic steatosis in the elderly [38]. A second hypothesis is the neuronal pathway from the liver might modulate peripheral insulin sensitivity [11]. A third hypothesis is that some nutrients, such as fatty acids and amino acids, might link hepatic steatosis and skeletal muscle IR [39]. A fourth hypothesis is that a liver-derived hormone (a hepatokine) affects the distant organ insulin sensitivity. We previously isolated hepatokine selenoprotein P, which is overproduced under an overnutrition state and causes IR both in the liver and skeletal muscle [13]. In addition, serum levels of selenoprotein P are inversely associated with serum levels of adiponectin [40] that enhance skeletal muscle insulin sensitivity [12]. Therefore, overproduction of selenoprotein P in association with hepatic steatosis, by directly or indirectly lowering adiponectin levels, causes skeletal muscle IR.

There are several limitations to this study. First, this was an observational study, and we were unable to examine causal associations. A large-scale longitudinal study is needed to clarify whether hepatic steatosis is a consequence or cause of skeletal muscle IR. Second, many of the study subjects had glucose intolerance/diabetes, although the severity was relatively mild as shown by the OGTT. Therefore, IR of each organ was possibly greater in our study subjects than in the general population, which could have influenced the results. Third, fifteen out of 69 subjects were taking metformin which might influence hepatic glucose production. However, major study results were similar in diabetic subjects, non-diabetic subjects, and subjects without metformin (data not shown). Fourth, we did not collect arterial or arterialized blood samples to perform the insulin clamp because these were not included in the manufacturer’s protocol of the artificial pancreas model STG-55. Further study should be required to confirm our conclusion by using arterial or arterialized blood samples.

In summary, the present study revealed an unexpected lack of an association between fat and local organ-specific IR in the skeletal muscle and adipose tissue. Instead, liver fat is strongly associated with skeletal muscle IR as well as with liver IR, suggesting a central role of fatty liver in the development of IR and that a network exists between liver and skeletal muscle to maintain whole-body energy homeostasis.

## Supporting Information

Figure S1 **Correlation between ectopic fat and insulin resistance (IR) in the liver, skeletal muscle, and adipose tissue.** Liver fat (steatosis score) was associated with skeletal muscle IR index (Rd) as well as with IR in the liver (HGP×FPI). Intramyocellular lipid was not associated with skeletal muscle IR index (Rd). Total fat mass was associated with HGP×FPI and Rd, but not with adipose tissue IR index (%FFA).
(PDF)

Table S1 **Multiple regression models predicting HGP×FPI and Rd in subjects without type 2 diabetes (** ***n*** **= 32).** HGP, hepatic glucose production; FPI, fasting plasma insulin Model 1, adjusted for, age, sex, and body mass index; Model 2, adjusted for, age, sex, body mass index, and total fat mass.
(DOC)

Table S2 **Multiple regression models predicting HGP×FPI and Rd in subjects with type 2 diabetes (** ***n*** **= 37).** HGP, hepatic glucose production; FPI, fasting plasma insulin Model 1, adjusted for, age, sex, and body mass index; Model 2, adjusted for, age, sex, body mass index, and total fat mass.
(DOC)
