# Supplementary figures and images for: Ectopic Fat Accumulation and Distant Organ-Specific Insulin Resistance in Japanese People with Nonalcoholic Fatty Liver Disease
Source: PLoS One. 2014 Mar 20;9(3):e92170. doi: 10.1371/journal.pone.0092170 (PMC3961287; doi:10.1371/journal.pone.0092170)

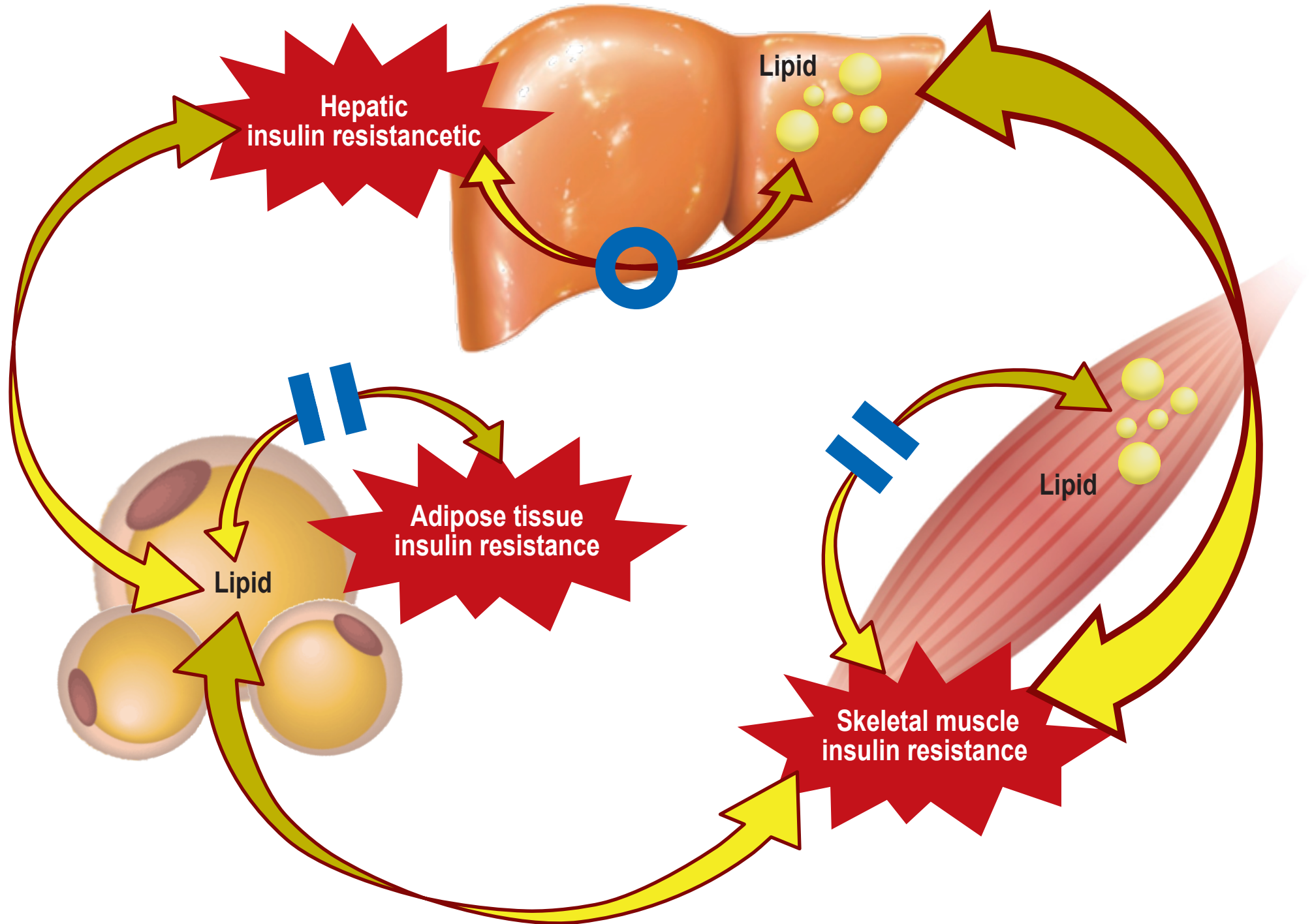

Supplement: Figure S1 — Correlation between ectopic fat and insulin resistance (IR) in the liver, skeletal muscle, and adipose tissue. Liver fat (steatosis score) was associated with skeletal muscle IR index (Rd) as well as with IR in the liver (HGP×FPI). Intramyocellular lipid was not associated with skeletal muscle IR index (Rd). Total fat mass was associated with HGP×FPI and Rd, but not with adipose tissue IR index (%FFA). (PDF) [file pone.0092170.s001.pdf]
