# Supplementary material for: Ectopic Fat Accumulation and Distant Organ-Specific Insulin Resistance in Japanese People with Nonalcoholic Fatty Liver Disease
Source: PLoS One. 2014 Mar 20;9(3):e92170. doi: 10.1371/journal.pone.0092170 (PMC3961287; doi:10.1371/journal.pone.0092170)
Supplement: Table S1 — Multiple regression models predicting HGP×FPI and Rd in subjects without type 2 diabetes ( n = 32). HGP, hepatic glucose production; FPI, fasting plasma insulin Model 1, adjusted for, age, sex, and body mass index; Model 2, adjusted for, age, sex, body mass index, and total fat mass. (DOC) [file pone.0092170.s002.doc]

Table S1 (Supporting Information) －Multiple regression models predicting HGP × FPI and Rd in subjects without type 2 diabetes (*n* = 32).

HGP, hepatic glucose production; FPI, fasting plasma insulin

Model 1, adjusted for, age, sex, and body mass index; Model 2, adjusted for, age, sex, body mass index, and total fat mass.
